# Subclavian Vein Stenosis Imitating Inflammatory Breast Cancer

**DOI:** 10.7759/cureus.32184

**Published:** 2022-12-04

**Authors:** Alex C Judd, Craig Weinkauf, Jennifer Erdrich

**Affiliations:** 1 Department of Surgery, University of Arizona, Tucson, USA

**Keywords:** inflammatory breast cancer, benign breast condition, lymphedema, peau d'orange, subclavian vein stenosis

## Abstract

Unilateral breast erythema, edema, and *peau d’orange* are classically associated with inflammatory breast cancer. However, occasionally this constellation of symptoms is seen with other causes. Maintaining a broad differential can therefore save a prospective patient from months of worry about a possible cancer diagnosis, untreated symptoms, and unnecessary and expensive tests. Here we present the case of a 75-year-old woman with a history of pacemaker placement complicated by left upper extremity deep venous thrombosis (DVT) who subsequently developed left breast *peau d’orange*, swelling, and erythema. After initially being worked up for inflammatory breast cancer, including multiple breast biopsies, she was then referred to specialists in cardiology, allergy, pulmonology, rheumatology, dermatology, lymphedema therapy, and vascular surgery undergoing an exhaustive workup that spanned nearly a year. Eventually, a venogram was performed, which revealed complete occlusion of her left subclavian vein. After undergoing angioplasty and stenting, her symptoms resolved.

## Introduction

Unilateral breast erythema, edema, and *peau d’orange* are common symptoms of inflammatory breast cancer [1,2]. However, the same symptoms are also sometimes seen in central vein stenosis, most commonly as a result of catheterization for dialysis [3,4]. We present a case in which a woman developed unilateral breast edema, erythema, and *peau d’orange*, undergoing a long and laborious workup before being diagnosed with subclavian vein occlusion. Following angioplasty and stenting, her symptoms resolved. A possible cause for the subclavian vein stenosis, in this case, is the placement of a dual chamber pacemaker and subsequent development of ipsilateral upper extremity deep venous thrombosis (DVT).

## Case presentation

**Figure 1:**
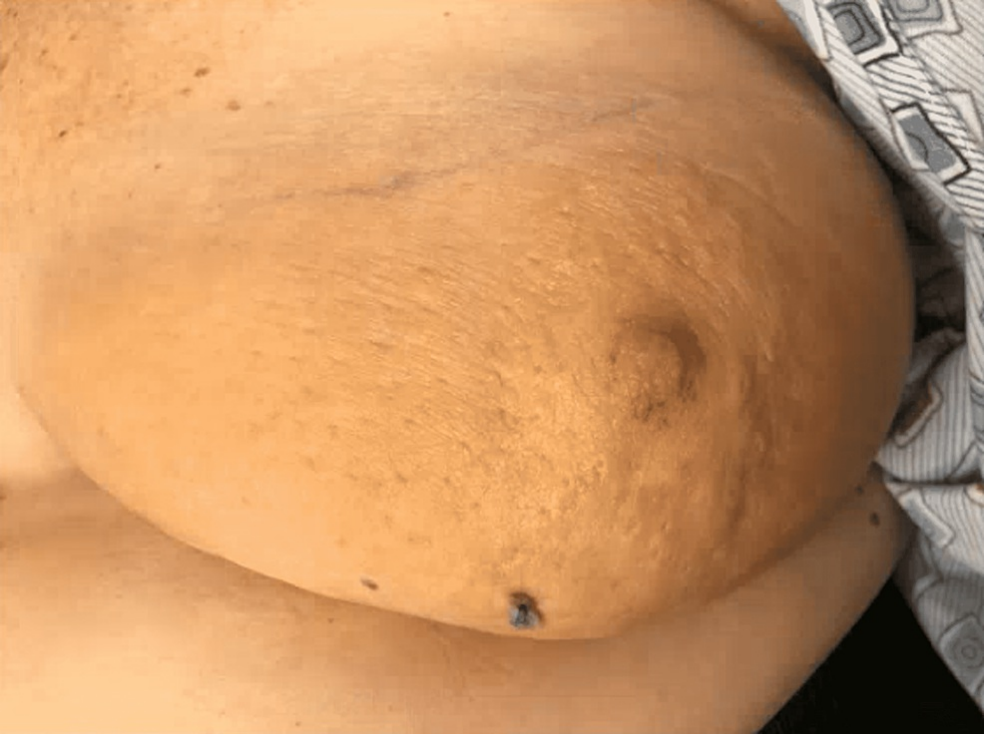
Left breast While no photo was taken at the height of the erythema, this one taken after partial resolution is still notable for edema and “*peau d’orange*” appearance.

The patient is a 75-year-old woman with a history of gout, hypertension, hypothyroidism, abdominal aortic aneurysm, paroxysmal atrial fibrillation, sick sinus syndrome (SSS), dual chamber pacemaker placement in 2015, left upper extremity DVT, and angioedema who presented with left breast warmth, pain, edema, and erythema (Figure 1). She initially presented at an outside institution and made her way to three other specialists before finally arriving at our clinic.

Of note, the patient underwent pacemaker placement for SSS five years prior to presenting to our clinic. Shortly after pacemaker placement, she developed left arm pain and swelling and was diagnosed with DVT. The pacemaker recorded paroxysmal atrial fibrillation so the patient was started on warfarin, later transitioning to apixaban. Approximately four months prior to the onset of her breast symptoms, the patient met with her cardiologist. A review of her pacemaker data revealed a very low ongoing atrial fibrillation burden and the decision was therefore made to stop her apixaban.

**Figure 2:**
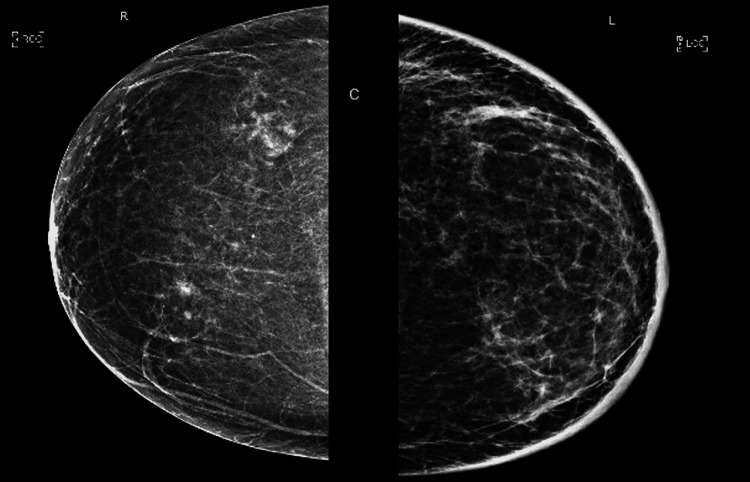
Mammogram revealed thickened skin and increased vascular markings of the left breast

**Figure 3:**
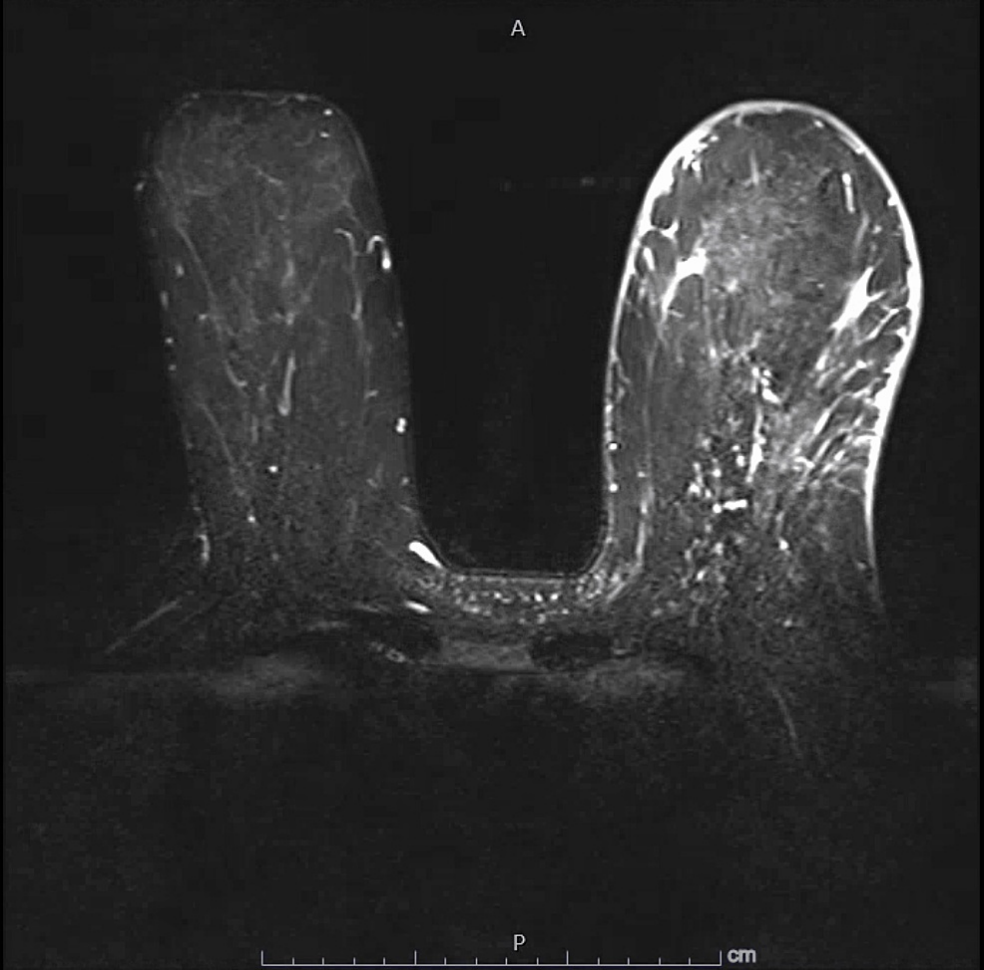
Breast MRI again revealed significant skin thickening and vascularity of the left breast

The patient reports that she first noticed erythema of her left breast, most prominent in the morning, which progressively worsened and was soon accompanied by edema and *peau d’orange*. At first, she endorsed breast heaviness and discomfort without outright pain and denied nipple discharge. She was evaluated by her primary care provider (PCP) who prescribed a course of triamcinolone cream and Augmentin for suspected mastitis. This treatment did not provide any relief. Her PCP then sent the patient for a left breast ultrasound (US) and diagnostic mammogram (MMG). The US revealed edema, prominent skin thickening up to 0.5 cm throughout the breast, and increased vascularity. MMG also demonstrated skin thickening as well as increased vascular markings (Figure 2). This prompted a referral to breast surgery for evaluation of possible inflammatory breast cancer. A punch biopsy was performed and revealed mild perivascular inflammation but was negative for malignancy. A breast MRI was performed and demonstrated marked skin thickening and increased vascularity without evidence of underlying malignancy (Figure 3). The patient sought out additional opinions from a community breast surgeon and a community dermatologist, each of whom performed additional punch biopsies. One of the biopsies noted dermal sclerosis, but both were once again negative for malignancy. The patient was sent to her cardiologist due to concern that the pacemaker might be responsible for her symptoms. The cardiologist ordered a duplex of the left upper extremity, which was negative for DVT, and an echocardiogram, which was normal.

**Figure 4:**
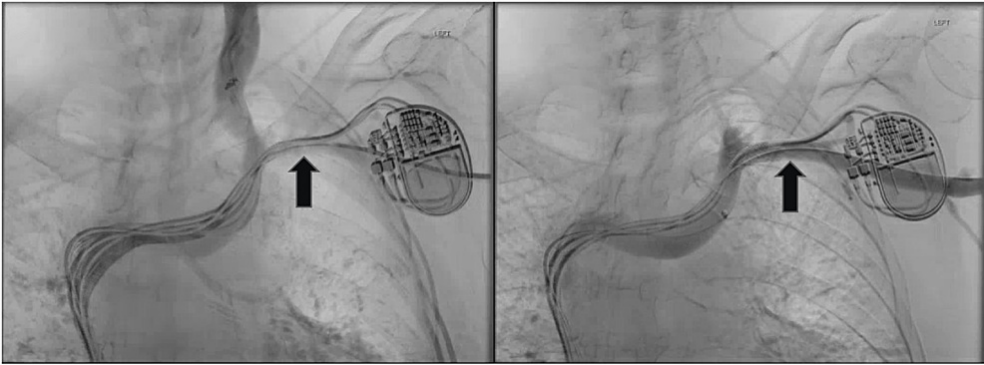
Venogram revealed high-grade stenosis near the confluence of the left subclavian and internal jugular veins, identified by the arrow on the left. The arrow on the right demonstrates improved flow through the same area after stent placement.

With worsening symptoms and still without a diagnosis, the patient then arrived at our clinic in search of yet another opinion. In addition to ongoing edema, erythema, and *peau d’orange*, she now endorsed pain. Crucially, our clinical exam was also notable for the interval development of dilated superficial veins and telangiectasias concerning for vascular congestion. This raised suspicion of a potential vascular etiology and vascular surgery was consulted. The left upper extremity duplex was repeated, and while there was no evidence of DVT, there was a notable absence of expected phasicity of flow in the subclavian vein, concerning for occlusion. Because of this, the patient went to the OR and underwent a venogram. This revealed abrupt occlusion of the left subclavian vein near the confluence with the left internal jugular vein with significant collateral flow observed (Figure 4). Despite repeated balloon angioplasty, high-grade stenosis persisted. Therefore, a 7 mm x 29 mm stent was placed across the lesion and dilated to 10 mm. After surgery, the patient was started on Plavix.

In the immediate postoperative period, the patient kept appointments from previous referrals. She saw an allergist to assess if an allergy to one of the pacemaker components might be contributing to her symptoms. Patch testing revealed an allergy to nickel but not to any of the listed components of the patient’s device. She was also referred to rheumatology and dermatology to evaluate for possible morphea or other connective tissue disorders, given the previously observed dermal sclerosis. The punch biopsy was repeated for the fourth time and was not consistent with morphea.

When she next presented to our clinic approximately a month after surgery, the erythema was notably absent. The edema and subjective discomfort continued at first, but these also demonstrated slow but gradual improvement in the ensuing weeks to months. The patient also saw a lymphedema therapist in the postoperative period and felt that the physiotherapy helped contribute to her improvement. By four months postoperatively, the remainder of the patient’s symptoms had resolved.

## Discussion

It is not surprising that initially, the patient’s symptoms were mistaken for inflammatory breast cancer (IBC), given the presenting symptoms of unilateral breast erythema, edema, and *peau d’orange*. These clinical findings are, of course, classically associated with IBC, which represents a “can’t miss” diagnosis as the deadliest of all breast cancer variants. IBC accounts for about 2.5% of all breast cancer diagnoses and 7% of all breast cancer deaths. Skin changes, such as erythema, edema, and *peau d’orange*, typically progress rapidly and must cover at least one-third of the breast to meet diagnostic criteria. Skin thickening on breast imaging is another finding from this case that is commonly seen in IBC. The diagnosis is confirmed by finding evidence of invasive carcinoma; sometimes, a disparate mass is discovered on imaging, and other times, tumor emboli are seen invading dermal lymphatics on punch biopsy. IBC is oftentimes confused for cellulitis or mastitis at first, though the rapid progression of symptoms and the lack of response to antibiotics generally prompt reconsideration [1,2].

It is difficult to definitively identify the source of the patient’s subclavian vein stenosis. However, the history of pacemaker placement is salient. The placement of cardiac implantable electronic devices, including pacemakers, has long been a known risk factor for the development of central vein stenosis. This association has been consistently demonstrated in studies dating as far back as the 1970s [5,6]. The most likely mechanism is that the implanted devices cause local tissue trauma and associated inflammation which then lead to the formation of microthrombi, intimal hyperplasia, and fibrosis [3]. A recent review by Rozmus et al. compiled a list of 7 articles that included a total of over 700 patients in which the composite incidence of abnormal venogram following pacemaker or implantable cardioverter defibrillator placement was 38%. Individual studies reported an incidence that ranged from 23-64%, which varied primarily based on the severity of stenosis required to be included and the length of follow-up. However, only 3% of patients experienced clinically significant symptoms [7]. Other studies have found that extensive collateral venous circulation is often observed at the time of diagnosis and this is thought to explain the low rate of overt symptoms [8]. The most common symptoms are ipsilateral arm and face edema. A history of previous DVT and the presence of multiple leads may represent risk factors for developing symptoms, though studies have thus far come to mixed conclusions [9]. Unilateral breast edema has been described as a rare complication of central vein stenosis (CVS) secondary to central venous catheterization, especially for hemodialysis, but there are currently no similar reports of asymmetric breast edema following pacemaker placement [4,10]. Of note, there have also been case reports of patients developing lymphedema, either alone or alongside associated central vein stenosis, following pacemaker placement [11]. Lymphedema was one of the manifesting symptoms of our patient’s central vein stenosis, and it is important to note this exam finding as a potential sign for the diagnosis of central vein stenosis for future patients. When this occurs, relieving the stenosis is a critical step of management but lymphedema physiotherapy can be a beneficial adjunct as well, as it was for our patient. The other key exam finding was the asymmetric superficial venous engorgement, which prompted a referral to vascular surgery. At that point, the patient had completed a duplex ultrasound, which was read as unremarkable, a study with 80-90% sensitivity and specificity for the diagnosis of central venous stenosis [12,13]. However, the exam finding raised the index of suspicion, demonstrating an instance when an exam finding in the appropriate clinical context supersedes imaging alone.

Our hope is that this case encourages a high index of suspicion for central vein stenosis in patients with unexplained unilateral breast edema, erythema, and dilated superficial veins. Once IBC has been ruled out, CVS should be considered, especially if the patient has a history of central vein catheterization or pacemaker placement. With a strong suspicion of CVS, angiography should be performed on the access and complete venous outflow tract. Ultrasound of central veins is difficult. Thus, if CVS is suspected in the absence of a clear source, additional imaging, such as chest X-ray, computed tomography, or MRI, can be helpful in evaluating possible external compression secondary to a mass, goiter, or aortic aneurysm [14]. Treatment consists of removing triggers if possible and then performing percutaneous transvenous angioplasty with or without stent placement. Radiological interventions are effective, and an open surgical approach is rarely required [15].

## Conclusions

Following an evidence-based, prompt workup for CVS as described above will avoid repeated breast biopsies and psychological harm associated with a looming potential cancer diagnosis. It will save the money and time associated with a drawn-out diagnostic process involving multiple referrals, procedures, and imaging studies. Most importantly, it will lead to prompt identification and treatment of the discomfort caused by breast erythema, edema, and *peau d’orange* not explained by IBC.
